# Evaluation of newborn sickle cell screening programme in England: 2010–2016

**DOI:** 10.1136/archdischild-2017-313213

**Published:** 2017-11-05

**Authors:** Allison Streetly, Rupa Sisodia, Moira Dick, Radoslav Latinovic, Kirsty Hounsell, Elizabeth Dormandy

**Affiliations:** 1 School of Population Health and Environmental Sciences, Faculty of Life Sciences & Medicine, King’s College London, London, UK; 2 Healthcare Public Health, Health Protection and Medical Directorate, Public Health England, London, UK; 3 Department of Paediatrics, Kings College Hospital, NHS Foundation Trust, London, UK; 4 PHE Screening, Health Improvement Directorate, Public Health England, London, UK

**Keywords:** screening, haematology, health services research, outcomes research, immunisation

## Abstract

**Objective:**

To evaluate England’s NHS newborn sickle cell screening programme performance in children up to the age of 5 years.

**Design:**

Cohort of resident infants with sickle cell disease (SCD) born between 1 September 2010 and 31 August 2015 and followed until August 2016.

**Participants:**

1317 infants with SCD were notified to the study from all centres in England and 1313 (99%) were followed up.

**Interventions:**

Early enrolment in clinical follow-up, parental education and routine penicillin prophylaxis.

**Main outcome measures:**

Age seen by a specialist clinician, age at prescription of penicillin prophylaxis and mortality.

**Results:**

All but two resident cases of SCD were identified through screening; one baby was enrolled in care after prenatal diagnosis; one baby whose parents refused newborn screening presented symptomatically. There were 1054/1313 (80.3%, 95% CI 78% to 82.4%) SCD cases seen by a specialist by 3 months of age and 1273/1313 (97%, 95% CI 95.9% to 97.8%) by 6 months. The percentage seen by 3 months increased from 77% in 2010 to 85.4% in 2015. 1038/1292 (80.3%, 95% CI 78.1% to 82.5%) were prescribed penicillin by 3 months of age and 1257/1292 (97.3%, 95% CI 96.3% to 98.1%) by 6 months. There were three SCD deaths <5 years caused by invasive pneumococcal disease (IPD) sensitive to penicillin.

**Conclusion:**

The SCD screening programme is effective at detecting affected infants. Enrolment into specialist care is timely but below the programme standards. Mortality is reducing but adherence to antibiotic prophylaxis remains important for IPD serotypes not in the current vaccine schedule.

**What is already known on this topic?**

- Newborn screening enables early identification of infants with sickle cell disease (SCD).
- Early entry into care aims to allow timely offer of penicillin prophylaxis and ensure that parents are aware of signs and symptoms of SCD.
- Introduction of conjugate pneumococcal vaccine in the universal immunisation programme has led to a reduction in morbidity and mortality in invasive pneumococcal disease.

**What this study adds?**

- The newborn screening programme accurately identifies babies with sickle cell disease.
- 80% of infants are enrolled into specialist care by 3 months of age and almost all are seen by 6 months of age.
- Mortality is now low in children with SCD under the age of 5 years but despite penicillin prophylaxis and conjugate pneumococcal vaccination, deaths still occur from invasive pneumococcal disease.

## Background

Without treatment, children with sickle cell disease (SCD) have high mortality from infection,^1^ splenic sequestration, anaemia and an increased risk of stroke.^2 3^ Infants are at increased risk of invasive pneumococcal disease (IPD) as well as infections due to early onset of functional hyposplenism. Early prophylactic penicillin,^4^ pneumococcal vaccination and parental education^5^ potentially reduce premature mortality.

Newborn screening enables early identification to ensure that affected babies enter care before they develop functional hyposplenism.^5 6^ In the USA, introduction of screening programmes and early intervention was associated with improved survival^6^ with over 94% of children reaching adulthood. The introduction of the pneumococcal conjugate vaccine (PCV) was followed by a marked reduction of fatal pneumococcal infections.^7–9^ In England, the introduction of PCV (in 2006 and 2013) and the conjugate Haemophilus vaccine in 1992 may have helped to reduce mortality in infants.^8^ Telfer *et al* reported low SCD mortality in East London although vaccine coverage is variable across England.^10 11^

The NHS newborn SCD screening programme—implemented in England between 2003 and 2006—aims to reduce SCD mortality and to minimise morbidity. This paper aims to evaluate the performance of the screening programme against the programme standards (table 1) and aligned clinical standards.^12^

**Table 1:** NHS Sickle Cell and Thalasaemia Screening Programme Standards, Second Edition, 2011

*Standard number*	*Objective*	*Acceptable Standard*	*Achievable Standard*
*NO1*	*Babies detected with sickle cell disease should have the best possible survival as assessed by mortality rate in children<5 years.*	*Four per 1000 person-years of life or 2 deaths per 100 affected babies.*	*Two per 1000 person-years of life or 1 death per 100 affected babies.*
*NO2i*	*Identify babies with disease with specified sensitivity and offer early intervention if required.*	*99% detection rate for Hb SS, 98% for Hb SC and 95% for other conditions.*	*99.5% detection rate for Hb SS, 99% for Hb SC and 98% for other conditions.*
*NO2ii*	*Coverage of screening test (tested/eligible)*	*95% of babies eligible for sickle cell screening receive a conclusive bloodspot screening test.*	*99% of babies eligible for sickle cell screening receive a conclusive bloodspot screening test.*
*NP4*	*All registered screen-positive babies are followed up and entered in care with a specialist/local centre.*	*90% seen by a specialist/local centre by 3 months of age; 95% reviewed annually by a specialist centre.*	*95% seen by a specialist/local centre by 3 months of age; 98% reviewed annually by a specialist centre.*
*NP6i*	*Ensure prophylactic treatment is offered and prescribed to screen-positive babies in a timely manner; offer parental education.*	*90% offered and prescribed Penicillin V or alternative by 3 months of age.*	*99% offered & prescribed Penicillin V or alternative by 6 months of age.*

## Methods

### National screening programme

Newborn bloodspot screening is offered to the parents/carers of all infants in England at 5–8 days of age and has included SCD since 2006. Verbal consent is obtained from a parent prior to the test and recorded in the parent-held record (https://www.gov.uk/guidance/newborn-blood-spot-screening-programme-overview).

Four methods of analysis are used for newborn screening using dried blood spot samples: (1) high performance liquid chromatography; (2) capillary electrophoresis; (3) tandem mass spectrometry and (4) isoelectric focusing are used for first-line screening. An alternative procedure using a different principle must be used for second-line testing to validate the result.^13^ All parents of a newly diagnosed infant are given a free copy of ‘A parent’s guide to managing your child with sickle cell disease’ in which the importance of adherence to penicillin prophylaxis and seeking urgent medical attention if a child has a persistent temperature >38.5°C is emphasised (https://www.gov.uk/government/uploads/system/uploads/attachment_data/file/408029/Sickle_Cell_A_ParentsGuide_2013.pdf).

### Study design and participants

We conducted a cohort study of all babies born with SCD in England between 1 September 2010 and 31 August 2015, followed up to 31 August 2016. Infants were registered by 13 centres undertaking second-level newborn screening of bloodspot samples, 18 centres providing community care for patients with SCD and 59 local hospitals and 18 specialist clinics. There were 1317 infants resident in England from birth with a diagnosis of SCD.

### Ethical considerations

An application under Section 251 was approved by the National Information Governance Board for the NHS Sickle Cell and Thalasaemia Screening Programme to collect named data without consent on all affected babies for a limited number of data items. This permission was reviewed and reissued on an annual basis. Cases that opted out of the programme were excluded from any further follow-up and identifiable information removed. The Sickle Cell Society and the UK Thalassaemia Society collaborated in and supported this application.

### Case ascertainment

Case ascertainment of study participants was obtained in several different ways as follows:

Group 1: babies with positive bloodspot test results reported by screening laboratories using a standard data collection form.^14^

Group 2: babies presenting symptomatically but not identified by screening. All newly diagnosed babies not identified by screening were notified to the project by specialist hospital centres.^15^

Group 3: babies from groups 1 and 2 who died. Mortality data obtained from the Office of National Statistics (ONS) and/or Medical Research Information Services and/or clinical networks.

Group 4: babies not identified until death, whose death records included International Statistical Classification of Disease and Health related codes linked to SCD or thalassaemia (ICD 10 codes D56 and D57), reported by ONS.

Clinics and community services also identified affected infants moving into or away from the area. All deaths were directly reported to the programme by clinicians as well (group 4). The case histories of all deaths were reviewed with postmortems and any other clinical information.

### Data collection

The dataset collected included screening results, demographic information and midwife-assigned ethnic group derived from the bloodspot card. Additional information included: confirmation of screening result; age first seen by a specialist clinician; parents’ antenatal screening history; age first prescribed penicillin (and initially vaccination status). The main outcome measures were: the proportion of affected babies offered newborn screening; the age seen by a specialist clinician as a measure of timely entry to care; the proportion of babies enrolled in clinic and prescribed penicillin by 3 months of age, completeness of the parent’s antenatal screening history; mortality up to the age of 5 years. These outcomes were compared against screening programme standards.^12^

### Statistical analysis

Statistical analyses were conducted using Stata 14,^16^ to evaluate the timeliness of visit to clinic and if required, prescription for penicillin. A Poisson’s model was employed to estimate the relative risk ratio for change in the number seen by 90 days based on the year in which the child was born.^17^ Models were adjusted for sickle cell type, location and whether the child was assigned to a specialist clinic.

## Results

### Participants

**Figure 1:**
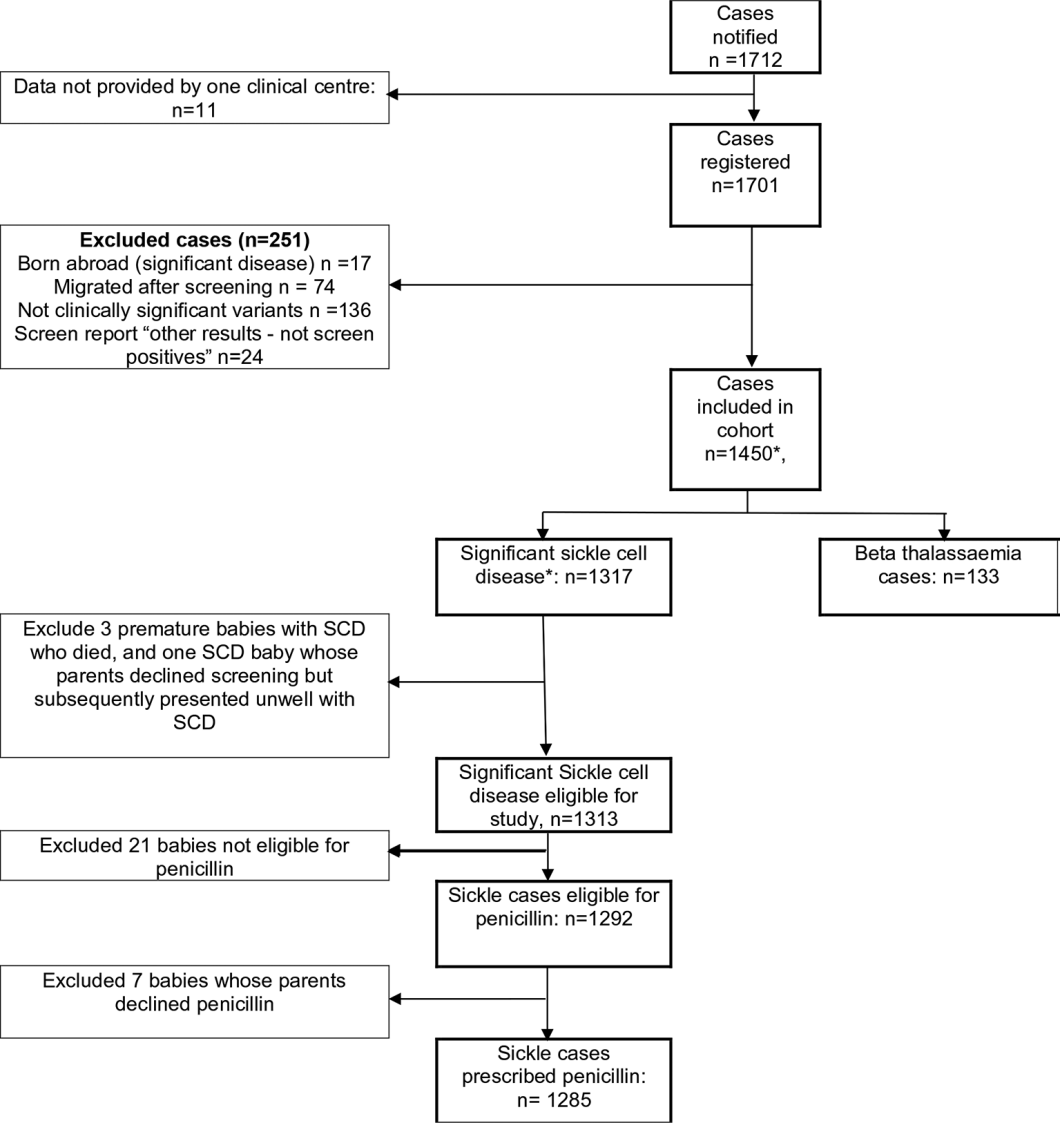
Flow chart of study particular. Includes three premature babies with sickle cell disease who died and one baby whose parents declined screening but subsequently presented unwell with sickle cell disease. *includes 3 premature babies with SCD who died, and 1 baby whose parents declined screening but subsequently presented unwell with SCD.

In total, 1712 cases were identified and 1701 registered with the programme (figure 1). One centre followed their own clinical governance procedures, did not accept the National Information Governance Board approval as data was not anonymised; 11 screen-positive cases from the study period were not included in the study.

Other exclusions are: 17 cases born abroad who moved into England at up to 1 year of age, 74 screen-positive cases who moved from England, 136 clinically insignificant haemoglobin variants; 24 non-screen-positive clinical variants reported. Data for one infant whose parents refused screening and later presented symptomatically and three babies who died before 3 months of age are excluded from the follow-up analysis. A total of 133 cases of beta thalassaemia are also excluded from further analysis.

All but 2 of the 1317 cases of SCD were detected by newborn screening. The parents of one affected infant declined screening because the risk status was already known from antenatal screening and prenatal diagnosis and one affected infant presented acutely ill following the parents declining newborn screening. All nine deaths were reported to the programme by both clinicians and the Office for National Statistics; all were found to have been screened. There were 4752 total years of follow-up for SCD cases.

Table 2 shows the breakdown of cases by disease type. The estimated birth prevalence for the study period for SCD is 0.39 per 1000 (1:2564).

**Table 2:** Types of sickle cell disease

*Condition*	*Freq.*	*(%)*
*Hb SS*	*853*	*(64.8)*
*Hb SC*	*366*	*(27.8)*
*Hb S/β+ thalassaemia*	*62*	*(4.7)*
*Hb S/HPFH*	*21*	*(1.6)*
*Hb S/β°thalassaemia*	*8*	*(0.6)*
*Other sickle type diseases*	*5*	*(0.4)*
*Hb S/δβ thalassaemia*	*<5*	* –*
*Total*	*1317*	*(100.0)*

*Figures are frequencies (column percentages). Other sickle type diseases include: Hb S/E, Hb S/Lepore and Hb S/Dpunjab. Percentages not shown for variants with frequency <5.*

### Timeliness of treatment

Table 3 shows that the timeliness of being seen in clinic has improved during the project; all cases were seen in clinic. 80.3% (95% CI 78% to 82.4%) of infants were seen by a clinician by 3 months of age and 97% (95% CI 95.9% to 97.8%) by 6 months. The proportion seen by 90 days increased from 221/287 (77%, 95% CI 71.7% to 81.7%) to 208/239 (85.4%, 95% CI 80.2% to 89.6%) with a statistically significant increase in the number being seen within 90 days of approximately 3% (2.9), (95% CI 1% to 4.8%) each year. Infants living in London were 115.6%), (95% CI 11.5% to 19.5%) less likely to be seen within the 90 days target while infants assigned to a specialist clinic were 11% (95% CI 6.6% to 19.2%) more likely to be seen by 90 days. There is no significant difference by the type of SCD (as compared with Hb SS).

**Table 3:** Timeliness of cases being seen in a specialist clinic

	*2010/11*		*2011/12*		*2012/13*		*2013/14*		*2014/15*		*Total*	
	*Freq.*	*%*	*Freq.*	*%*	*Freq.*	*%*	*Freq.*	*%*	*Freq.*	*%*	*Freq.*	*%*
*Seen in specialist clinic*												
*≤90 days*	*221*	*77.0*	*232*	*77.6*	*190*	*80.5*	*207*	*82.1*	*208*	*85.4*	*1054*	*80.3*
*91–120 days*	*42*	*14.6*	*31*	*10.4*	*26*	*11.0*	*26*	*10.3*	*22*	*9.2*	*147*	*11.2*
*121–150 days*	*12*	*4.2*	*20*	*6.7*	*13*	*5.5*	*6*	*2.4*	*4*	*1.7*	*55*	*4.2*
*151–180 days*	*3*	*1.0*	*6*	*2.0*	*2*	*0.8*	*4*	*1.6*	*2*	*0.8*	*17*	*1.3*
*Over 180 days*	*9*	*3.1*	*10*	*3.3*	*5*	*2.1*	*9*	*3.6*	*7*	*2.9*	*40*	*3.1*
*Grand total*	*287*	*100*	*299*	*100*	*236*	*100*	*252*	*100*	*239*	*100*	*1313*	*100*
Prescribed penicillin prophylaxis*												
*≤90 days*	*218*	*77.6*	*226*	*76.4*	*190*	*81.5*	*206*	*82.7*	*198*	*85.0*	*1038*	*80.3*
*91–120 days*	*43*	*15.3*	*35*	*11.8*	*29*	*12.4*	*24*	*9.6*	*20*	*8.6*	*151*	*11.7*
*121–150 days*	*12*	*4.3*	*21*	*7.1*	*6*	*2.6*	*5*	*2.0*	*4*	*1.7*	*48*	*3.7*
*151–180 days*	*3*	*1.1*	*9*	*3.0*	*3*	*1.3*	*4*	*1.6*	*1*	*0.4*	*20*	*1.5*
*>180 days*	*4*	*1.4*	*5*	*1.7*	*3*	*1.3*	*10*	*4.0*	*6*	*2.6*	*28*	*2.2*
*Declined*	*1*	*0.4*		*0.0*	*2*	*0.9*			*4*	*1.7*	*7*	*0.5*
*Grand total*	*281*	*100*	*296*	*100*	*233*	*100*	*249*	*100*	*233*	*100*	*1292*	*100*

*Figures are frequencies (column percentages).*

*Cases eligible for penicillin exclude S/HPFH and S Lepore.

In seven penicillin eligible cases (excluding S/HPFH and S Lepore), parents refused the offer of penicillin. 80.3% of cases (95% CI 78.1% to 82.5%) were prescribed penicillin by 3 months of age and 97.3% (95% CI 96.3% to 98.1%) by 6 months (table 3). A statistically significant increase in penicillin timeliness of approximately 2.6% (95% CI 0.7% to 4.6%) more new-borns being prescribed penicillin within 90 days of birth each year is reported. Location (London or elsewhere), type of SCD and whether the child had been allocated a specialist clinic were not associated with timeliness of penicillin prescribing.

Movers out are excluded from the main analysis due to lack of certainty about timing of movement but 64 out of 72 migrated babies had a record of a confirmed diagnosis; 41/57 (71.9%) with a SCD diagnosis were recorded as seen in clinic with one refusal of treatment noted.

### Predictive value of testing

Table 4 shows all cases with a screening and a confirmed result including the 1320 SCD screen-positive results, 154 clinically non-significant conditions and 64 babies with a confirmed diagnosis who have migrated. There were 1375 of 1447 screen-positive results that were confirmed as significant sickle cell conditions on repeat testing with 52 having Hb AS and 20 other not clinically significant conditions. Some suggest these cases would not be regarded as false-positive results even if not ‘true positives’ in clinical terms. The overall positive predictive value is 95% and specificity is >99% (approximately 3.25 million screens). No missed cases have been reported from testing during the period of the study or over the previous decade suggesting sensitivity near 100%.

**Table 4:** Cross-tabulation of screening result and confirmed diagnosis groupings

	*Confirmed diagnosis (groups)*												
*Screening Hb types present*	*Hb SS*		*Hb SC*		Sickle thalassaemias*		*Other sickle†*		*Hb AS*		*Other clinically insignificant*		*Total*
*F+S*	*886*	*90.4%*			*53*	*5.41%*	*24*	*2.45%*	*5*	*0.51%*	*12*	*1.22%*	*980*
*F+S+A*					*22*	*38.60%*			*35*	*61.40%*			*57*
*F+S+C*			*383*	*100%*									*383*
*F+S+D*							*3*	*100%*					*3*
*F+S+E*							*3*	*100%*					*3*
*F+S+other*			*1*	*4.80%*					*12*	*57.10%*	*8*	*38.10%*	*21*
*Total*	*886*		*384*		*75*		*30*		*52*		*20*		*1447*

*Sickle thalassaemias include the following conditions: Hb S/β+ thalassaemia, Hb S/β°thalassaemia and Hb S/δβ thalassaemia.

*†Other sickle is grouping all other sickle conditions: Hb S/E, Hb S/Lepore, Hb S/HPFH and Hb S/Dpunjab.*

### Mortality

There were nine deaths in children with SCD: five were under 1 year of age; four were aged between 1 and 3 years of age. Eight of the nine cases were Hb SS; none were Hb SC disease. Three were due to complications of prematurity, three others were born at term but died of causes unrelated to SCD. All three deaths ascribed to SCD occurred at over 1 year of age, all had positive blood cultures for *Streptococcus pneumoniae* (type 15 B sensitive to penicillin) and all had received Prevenar 13, one also had had pneumovax but two were under 2 years of age and so were not eligible. Two had associated acute splenic sequestration. The estimated infant mortality rate (deaths under 1 year) for all causes was 3.8/1000 comparable with the general population (none of the five deaths under 1 year were directly attributable to SCD). The death rates are 1.7/1000 person-years of follow-up for all sickle cell conditions and 2.6 per 1000 person-years for Hb SS alone.

Since this report was completed and after the end of the study period, one additional death has been reported attributed to strep pneumoniae septicaemia (type 12F) in a child who had not received Pneumovax (http://www.itv.com/news/central/2017-04-21/three-year-old-dies-after-gp-failed-to-give-him-life-saving-vaccine/).

## Discussion

### Main findings

The paper presents data for a cohort of children with sickle cell disorders based on the entire population of England. The data reveal good overall performance with coverage and test performance satisfactory with no known missed cases and only one refusal. Follow-up of babies with enrolment into clinics is good but, despite improvements in timeliness, one in five babies was not seen by a specialist clinician by 3 months of age, nor are they always prescribed penicillin by 3 months of age. Despite this, there were few deaths and mortality at 1.7 per 1000 person-years (2.6 per 1000 person-years for Hb SS) was similar or lower than other similar cohorts including: Netherlands (4.6/1000),^18^ New York 3.8/1000,^19^ Jamaica 3.1/1000^9^ and Belgium 2.5/1000.^20^ All deaths attributable to SCD over the age of 1 year were caused by penicillin-sensitive non-vaccine serotypes of IPD^21^ in line with US reports of a rise in non-vaccine-related bacteraemias.^22 23^ The difficulty of linking primary care with secondary care data highlights the challenge of data linkage to help prevent deaths in this vulnerable population.^24^ The importance of a focus on adherence^25^ and monitoring compliance through pharmacy records^26 27^ reviewing parental beliefs and reliable rapid access to prescriptions are emphasised,^24 27–29^ as is working with community groups noting seven cases where refusal to take penicillin, often reported as due to parents ‘putting all their faith in god’. Several of the deaths were in affected babies who were born prematurely, had interuterine growth retardation or other complications as noted elsewhere.^30^ ^31^

## Strengths and limitations

A key strength of the study is that data are from an almost complete national cohort. We acknowledge, however, that the cohort remains relatively small. While it is possible there may be missed cases, in practice, these are unlikely because the programme maintains high coverage and low refusal rates. We excluded ‘movers-out’ from the main analysis and have not presented detailed information on ‘movers-in’ but no deaths were recorded among those not identified through screening suggesting that the latter exclusion will not impact on conclusions. Any possible bias from under-reporting was minimised through notification to the study of all screening results without requiring consent, which was justified as it is known that there is a stigma associated with SCD and other haemoglobinopathies. Some parents deny their baby is affected; these babies are less likely to enter the care pathway and appear to have worse health outcomes. Only 2 out of the 1703 notifications were not screened (one of which was already known to be affected and the other presented symptomatically), so refusals are minimal.^12^ The national coverage of the bloodspot programme to residents is high and there are only 1000 refusals annually from 680 000 births screened (about 2/1000).^32^ 98.5% of babies have a conclusive result by 17 days and almost all by 6 weeks, so this is also unlikely to be a source of bias.^32^ We initially aimed to collect data on Prevenar (conjugate pneumococcal vaccine) uptake but this proved difficult due to lack of linkage with the information being held in primary care. As infants with SCD will have been offered this routinely (national coverage is 94% or 92% including booster at 12 months), most should have been immunised. In summary, the findings are likely to accurately represent the outcomes from the screening programme in England.

## Conclusion

The first evaluation of newborn screening programme established in England in 2006 shows promising outcomes and scope for improvement. Test performance and coverage appear excellent but timeliness of care, acceptance of penicillin and adherence are challenges. Delays between screening results and enrolment in care require optimisation of fail-safe follow-up. Deaths from non-vaccine penicillin sensitive pneumococcal infection emphasise the importance of support to parents, who may struggle to accept the news of the diagnosis of their baby and may need support with adherence to prophylaxis. It also emphasises the importance of education of the public and all front-line professionals of immediate access to and adherence to prophylaxis (penicillin and vaccination) and of seeking urgent medical attention if a child has a persistent temperature over 38.5°C. This may be a challenge in primary care given the focus on reducing antibiotic prescriptions.

Finally, the need continues to ensure the link from the specialised commissioning clinical networks governed by NHS England as part of its specialised services responsibility *including* clinical leadership which reaches out to local providers and parents to continue to educate, support and make accessible all available resources to help families cope with these challenging life-long conditions which continue to be stigmatised to ensure they have easy access to high-quality care, routinely reported on and to establish strong links from screening into the care pathway.
